# Genome sequence of *Bacillus anthracis* typing phage AP631

**DOI:** 10.1007/s00705-018-04135-3

**Published:** 2019-01-21

**Authors:** Xiankai Liu, Dongshu Wang, Chao Pan, Erling Feng, Hang Fan, Manli Li, Li Zhu, Yigang Tong, Hengliang Wang

**Affiliations:** 1 State Key Laboratory of Pathogens and Biosecurity, Beijing Institute of Biotechnology, 20 Dongdajie Street, Fengtai District, Beijing, 100071 China; 2 State Key Laboratory of Pathogens and Biosecurity, Institute of Microbiology and Epidemiology, 20 Dongdajie Street, Fengtai District, Beijing, 100071 China

## Abstract

AP631, a virulent bacteriophage of *Bacillus anthracis*, is widely used in China to identify anthrax bacteria. In this study, we report the complete AP631 phage genome sequence as well as comparative genomic analysis with other bacteriophages of *B. cereus* and related species. The double-stranded circular DNA genome of phage AP631 was 39,549 bp in length with 35.01% G + C content. The phage genome contained 56 putative protein-coding genes but no rRNA or tRNA genes. Comparative phylogenetic analysis of the phage major capsid proteins and terminase large subunits showed that phage AP631 belongs to the *B. cereus sensu lato* phage clade II. Comparative genomic analysis revealed a high degree of sequence similarity between phage AP631 and *B. anthracis* phages Wbeta, Gamma, Cherry, and Fah, as well as three AP631-specific genes bearing no significant similarity to those of other phages.

Phages that infect prokaryotes were first reported by Frederick W. Twort in 1915 [1]. Over time, bacteriophages have become useful tools for bacterial species and strain differentiation [2, 3]. In 1930, Cowles isolated the first *Bacillus anthracis* phage from crude sewage [4]. Since then, several additional phages of *B. anthracis* have been isolated from sources worldwide [5, 6], including the well-known gamma, phage which is currently used as a diagnostic tool to distinguish *B. anthracis* from other *B. cereus* group members [2]. Phage Fah was used widely in the former Soviet Union to identify anthrax bacteria [3]. In 1963, Dong et al. isolated a *B. anthracis* phage from crude sewage and designated it AP631, indicating that this was the first *B. anthracis* phage isolated in China [7]. Because AP631 can specifically infect non-encapsulated *B. anthracis* and form clear plaques, it is now widely used in China to identify anthrax bacteria. Under the electron microscope, AP631 has a hexagonal head around 50 nm in diameter and a long noncontractile tail around 180 nm in length [8]. Thus, AP631 could be taxonomically assigned to the family *Siphoviridae* in the order *Caudovirales*. In 2016, Zhang et al. found that AP631 could nonspecifically lyse eight isolates of *B. cereus* preserved in their laboratory, forming turbid plaques [9]. Molecular data on phage AP631 are not available. Here, we present the complete genome sequence of the AP631 bacteriophage and compare it with other phages of the *B. cereus* group. These data are useful for extending our understanding of the genomic characteristics of this group of phages and for enabling various applications related to the identification and control of *B. anthracis*.

## Bacterial strains and phage DNA isolation

Phage AP631 was obtained from the Lanzhou Institute of Biological Products Co., Ltd., Gansu Province, China, and propagated on *B. anthracis* strain A16R by plating on BHI agar, followed by amplification in BHI nutrient broth. Bacterial cells were removed from culture supernatants by filtration through a 0.22-μm syringe filter prior to isolation of bacteriophage genomic DNA. Genomic DNA was purified using a QIAGEN Lambda DNA Extraction Kit (QIAGEN, Germany) following the manufacturer’s instructions.

## Genome sequencing and annotation

Whole-genome sequencing was performed at the Beijing Novogene Bioinformatics Technology Co., Ltd. on the Illumina HiSeq PE150 platform with approximately 1340-fold coverage. High-quality paired-end reads were assembled *de novo* using SOAP [10, 11] (http://soap.genomics.org.cn/soapdenovo.html). Potential open reading frames (ORFs) were identified using PHASTER (http://phaster.ca) [12]. Potential tRNAs were identified using tRNAscan-SE [13] (http://lowel.ab.ucsc.edu//tRNAscan-SE/). Comparative analysis of phage AP631 nucleotide and amino acid sequences with other known sequences was performed using BLAST (http://blast.ncbi.nlm.nih.gov/Blast.cgi). Comparative phylogenetic analysis was conducted using the neighbor-joining method in MEGA6 [14] and ClustalW [15]. Genomic comparisons between the AP631 and reference genome sequences (or among two or more genomes) were conducted using local BLAST+ tools, and the alignment results were visualized using Easyfig2.2.3 [16] (http://easyfig.sourceforge.net/).

## The general genomic features of AP631

**Fig. 1:**
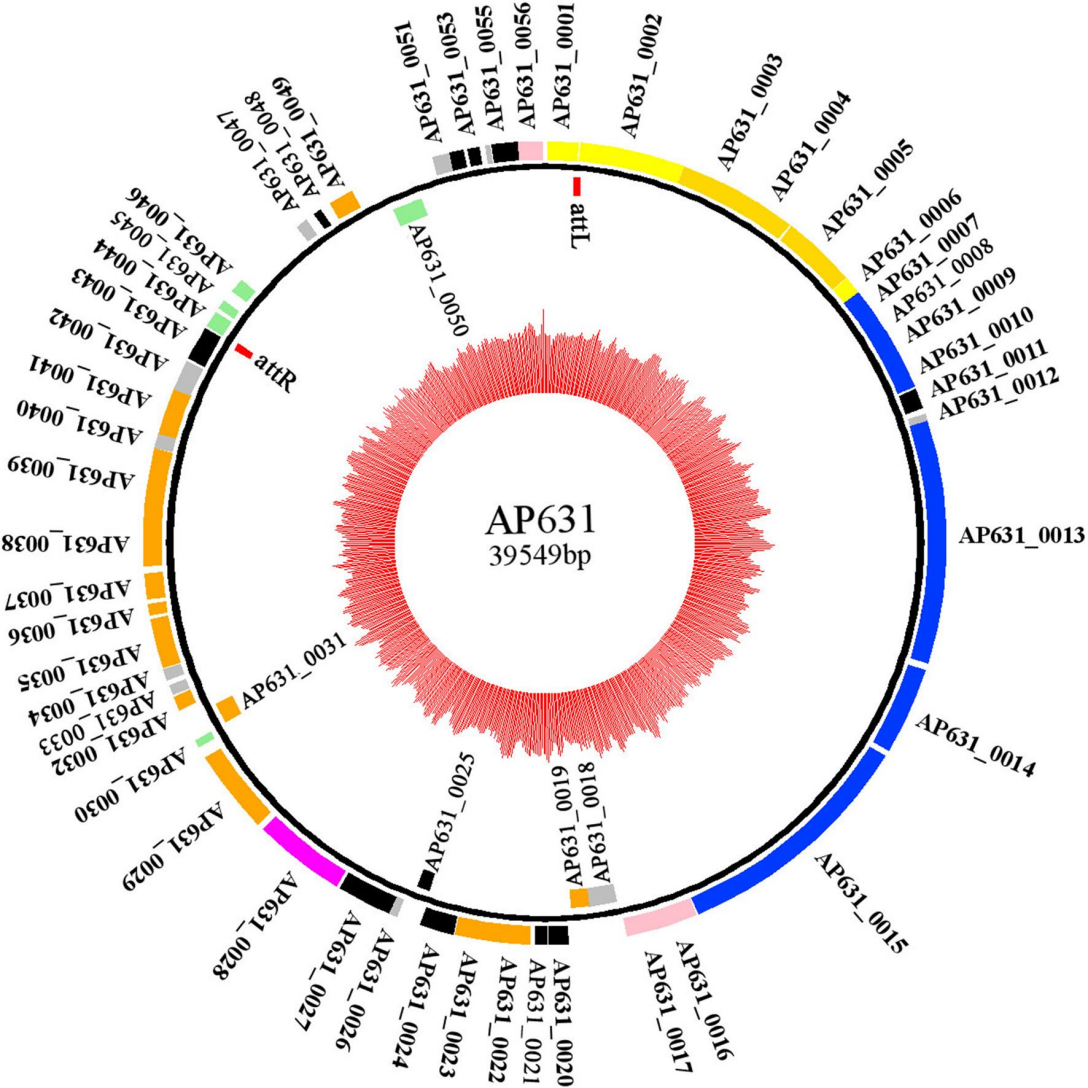
Circular representation of the phage AP631 genome. The innermost circle indicates the GC content (red). The outer circle indicates predicted ORFs located on the positive DNA strand, and the circle inside of that indicates predicted ORFs located on the negative DNA strand as well as the two att sites. Categories of functional ORFs are indicated by the following colors: att site, red; head protein, gold; tail protein, blue; DNA packaging, yellow; host cell lysis, pink; lysogeny control, magenta; DNA replication and gene expression, orange; conserved phage protein, black; hypothetical phage protein, gray; bacterial hypothetical protein, light green

The genome of phage AP631 is 39,549 bp in length with a G + C content of 35.01%, similar to that of its host *B. anthracis*. A total of 56 ORFs were identified as probable protein-coding genes. of which 51 were located on the positive strand, while only five ORFs were located on the negative strand. No rRNA or tRNA genes were identified (Fig. 1). Similar to most phage genomes, the AP631 genome was tightly packed: approximately 89% of the genome sequence encoded gene products, and on average, there were 1.42 genes per kb. Of the 56 ORFs, 51 (91%) shared a high degree of sequence similarity with predicted ORFs of *B. cereus* group phages, such as the *B. anthracis* phages Wbeta, Gamma, Cherry, and Fah. However, five ORFs showed no matches in the Virus and Prophage Database but had hits against the Bacterial Database or GenBank. Three of these five ORFs (ORF44–ORF46) were continuous and adjacent to the attR site. ORF44–ORF46 showed sequence similarity to genes of three different *Bacillus* species (*B. cytotoxicus*, *B. cereus* and *B. anthracis*, respectively), which probably indicates the evolutionary history and former hosts of phage AP631. The endolysin sequence of phage AP631 was identical to that of the *B. anthracis* phages Wbeta, Gamma, Cherry, and Fah.

## Phylogenetic analysis

**Fig. 2:**
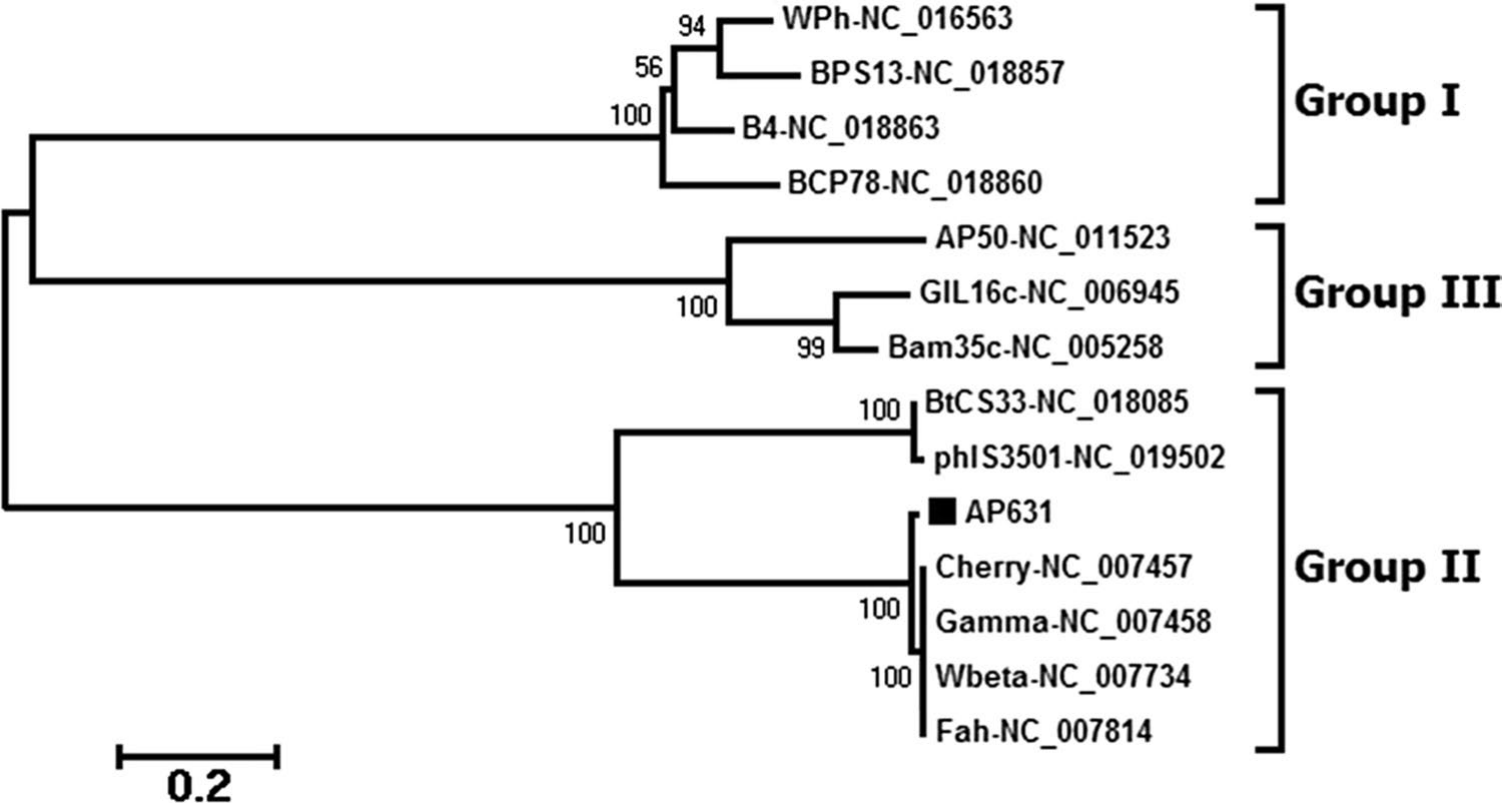
Comparative phylogenetic analysis. Comparative phylogenetic analysis of phage major capsid proteins and the terminase large subunits was performed using the neighbor-joining method in MEGA6 and ClustalW. Numbers associated with each branch represent bootstrap values

Genome sequences from 13 other *B. cereus* group phages were obtained from the NCBI in GenBank format. A phylogenetic analysis of the major capsid proteins and the terminase large subunits of all 14 phages (including AP631) was conducted using the neighbor-joining method in MEGA6 [14] and ClustalW [15] (Fig. 2). ClustalW was used to align the sequences, and MEGA6 was used to construct a neighbor-joining tree with 1,000 bootstrap replicates. This tree showed that phage AP631 is highly homologous to the *B. anthracis* phages Cherry, Gamma, Wbeta and Fah, which all belong to evolutionary group II (Fig. 2).

## Comparative genomic analysis

**Fig. 3:**
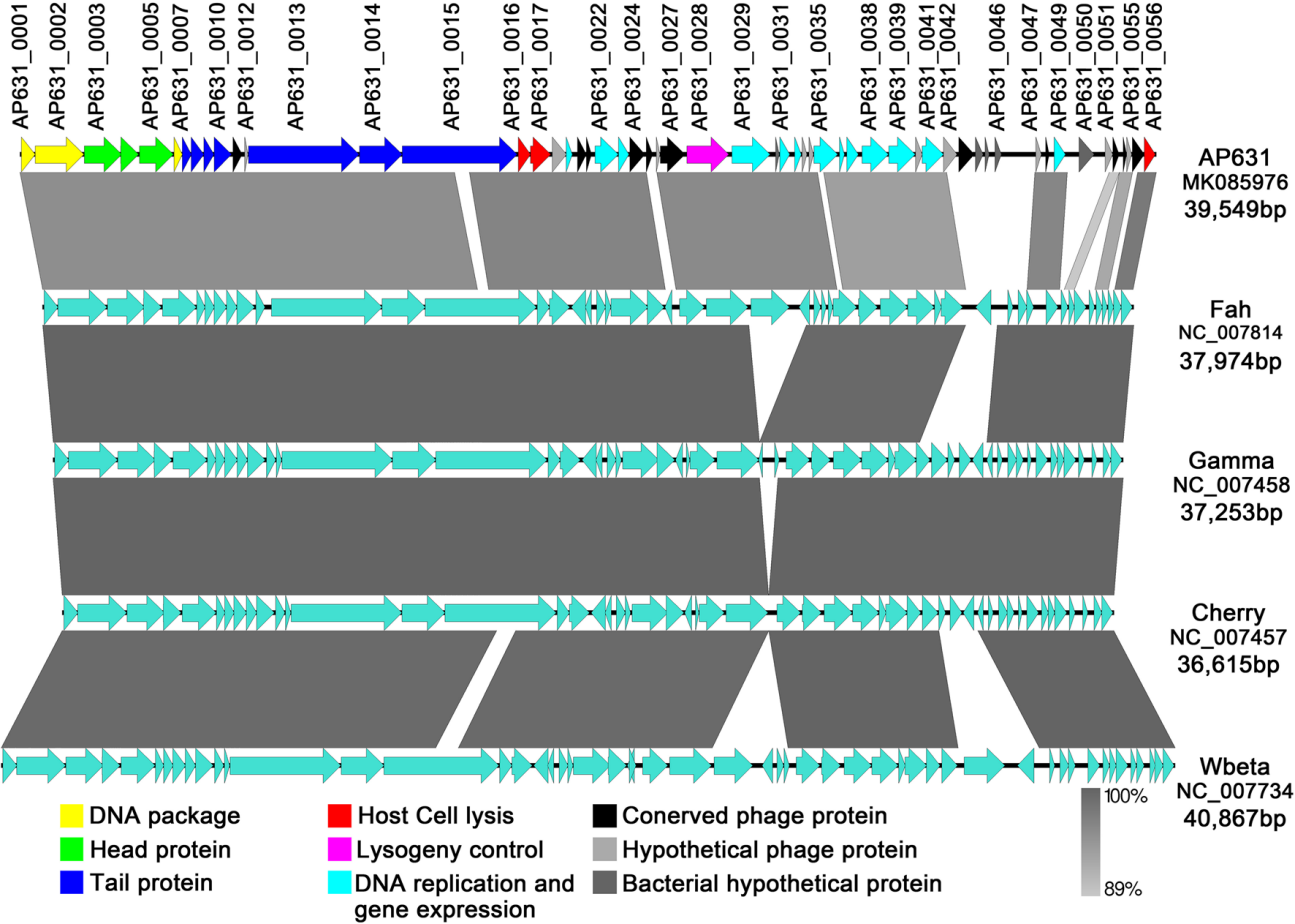
Comparison of the genome sequence of phageAP631 with those of the *B. anthracis* phages Cherry, Gamma, Wbeta and Fah. Predicted ORFs and the direction of transcription are indicated by block arrows. For phage AP631, ORFs are colored according to gene function, as indicated in the legend at the bottom. Conserved regions are shaded in grey, with the color intensity indicating the nucleotide sequence identity level (from 89% to 100%). Genomic comparisons were performed using BLASTn, and similarities with E values lower than 0.00001 were plotted. The figure was produced using Easyfig 2.2.3 [16] using data extracted from GenBank annotations

The genome size of phage AP631 is similar to those of the *B. anthracis* phages Cherry, Gamma, Wbeta and Fah, which range from 36,615 bp to 40,867 bp. The AP631 genome is colinear with the other four *B. anthracis* phage genomes and shows a similar cassette organization. The left end of the genome contains genes related to DNA packaging, the phage head protein, the head-tail adaptor protein, and tail proteins, and the central region of the genome contains genes related to host cell lysis, lysogeny control, DNA replication and gene regulation. These regions were found to be almost identical to those of the phage Fah genome and showed a high degree of similarity to the genomes of phages Gamma, Cherry and Wbeta. By contrast, the right end of the AP631 genome was highly divergent when compared with the other four *B. anthracis* phage genomes, and most of the genes in this region had no known function. Three ORFs (ORF44–ORF46), which showed no significant similarity to any proteins in the other four phage genomes, were located in the highly variable right genomic region (Fig. 3).

**Nucleotide sequence accession number:** The GenBank accession number for phage AP631 is MK085976.
